# High Concentration Intrinsic Defects in MnSb_2_Te_4_

**DOI:** 10.3390/ma16155496

**Published:** 2023-08-07

**Authors:** Jie Xiong, Yin-Hui Peng, Jia-Yi Lin, Yu-Jie Cen, Xiao-Bao Yang, Yu-Jun Zhao

**Affiliations:** Department of Physics, South China University of Technology, Guangzhou 510640, China; 17354426785@163.com (J.X.); pengyinhui88@gmail.com (Y.-H.P.); h752917623h@163.com (J.-Y.L.); cenyjie@gmail.com (Y.-J.C.); scxbyangscut@scut.edu.cn (X.-B.Y.)

**Keywords:** first-principles calculation, van der Waals material, MnSb_2_Te_4_, intrinsic defect, magnetic phase transition

## Abstract

MnSb_2_Te_4_ has a similar structure to an emerging material, MnBi_2_Te_4_. According to earlier theoretical studies, the formation energy of Mn antisite defects in MnSb_2_Te_4_ is negative, suggesting its inherent instability. This is clearly in contrast to the successful synthesis of experimental samples of MnSb_2_Te_4_. Here, the growth environment of MnSb_2_Te_4_ and the intrinsic defects are correspondingly investigated. We find that the Mn antisite defect is the most stable defect in the system, and a Mn-rich growth environment favors its formation. The thermodynamic equilibrium concentrations of the Mn antisite defects could be as high as 15% under Mn-poor conditions and 31% under Mn-rich conditions. It is also found that Mn antisite defects prefer a uniform distribution. In addition, the Mn antisite defects can modulate the interlayer magnetic coupling in MnSb_2_Te_4_, leading to a transition from the ideal antiferromagnetic ground state to a ferromagnetic state. The ferromagnetic coupling effect can be further enhanced by controlling the defect concentration.

## 1. Introduction

In recent years, MnSb_2_Te_4_, along with related systems, have received extensive attention. It can be regarded as a structure formed by inserting a layer of binary compounds, MnTe, into Sb_2_Te_3_, i.e., a septuple layer (SL) of Te-Sb-Te-Mn-Te-Sb-Te stack composition with a space group of $R\bar{3}m$. Mn^2+^ ions contribute approximately 5 *μ*_B_ magnetic moments each to the system, exhibiting interlayer antiferromagnetic (AFM) and intralayer ferromagnetic (FM) properties. Many quantum effects have been experimentally observed on MnBi_2_Te_4_, such as AFM topological insulators, axion insulator states, Chern insulator states, and Quantum anomalous Hall effects [1,2,3,4,5]. In addition, the Weyl semimetal state and flat Chern band are predicted in MnBi_2_Te_4_ [6,7,8]. In an analogy to MnBi_2_Te_4_, MnSb_2_Te_4_ has also attracted much attention. MnSb_2_Te_4_ was considered a trivial AFM insulator [6,9], but a recent density functional theory (DFT) study has shown that it is a nontrivial topological insulator [10]. The different performance of MnSb_2_Te_4_ under various synthesis conditions is likely attributed to the intrinsic defects in the samples. Interestingly, there is a conflict in the synthesis conditions for achieving the topological properties in MnSb_2_Te_4_. MnSb_2_Te_4_ achieves FM topological insulators with a few percent Mn excess [11]. However, in prior research, the decrease of Sb content at Mn sites led to an enhancement of ferrimagnetism, and the decrease of Mn concentration at Sb sites preferred a nontrivial band topology [12].

Prior experimental and theoretical work has revealed that there are Mn_Bi_, Bi_Mn_, and Bi_Te_ antisite disordered defects in MnBi_2_Te_4_ crystals [13]. These native defects have been demonstrated to change the properties of this material dramatically. For instance, high-concentration intrinsic Mn-Bi exchange defects and the formation of tellurium vacancies on the surface can lead to surface collapse in MnBi_2_Te_4_ [14]. DFT calculations elucidated that the lattice mismatch between MnTe and Bi_2_Te_3_ engenders a propensity for the formation of mixed Mn-Bi sites [15]. Additionally, as an analog of MnBi_2_Te_4_, MnSb_2_Te_4_ has similar types of intrinsic defects [15]. Single-crystal neutron diffraction and electron microscopy studies show a nearly random distribution of the antisite defects in MnSb_2_Te_4_ [16]. Due to the small difference in ion size and electronegativity between Mn and Sb, it was reported that MnSb_2_Te_4_ synthesized in the experiment has a high defect concentration, e.g., 15% [16] or 19.3% [17] Mn antisite defects on the Sb site. Generally, it is known that the concentration of defects largely depends on growth conditions, thereby affecting the properties of materials. For instance, Bi_Mn_ antisite defects are predicted to readily form under Te-poor conditions in MnBi_2_Te_4_ [15]. Sb antisite defects and Sb vacancies can form easily at less Te-rich conditions in Sb_2_Te_3_, which is confirmed by DFT calculations and experiments [18]. Therefore, the impact of the chemical potential environment on the formation of defects merits in-depth investigation.

Extensive prior work has explored the impact of defects on MnSb_2_Te_4_. The calculated formation energy of Mn antisite defects on the Sb sites was reported to be negative [15], indicating that the system might be intrinsically unstable as the defect could form spontaneously. MnSb_2_Te_4_ samples, however, can be synthesized in the experiment, in contrast to the calculated negative formation energy of Mn antisite defects. To clarify this, the stability of these intrinsic defects should be further studied. Intrinsic defects have a wide range of concentrations, depending on the growth conditions.

In this article, we have systematically investigated the intrinsic defects of MnSb_2_Te_4_ and their impact on the system. It is found that the Mn antisite defects are the intrinsic defects with the lowest formation energy, which will increase as the concentration increases. We find that the concentration of Mn antisite reaches around 15% at Mn-poor/Sb, Te-rich conditions under thermal equilibrium, and about 31% at Mn-rich/Sb, Te-poor conditions. Furthermore, it reveals that the Mn antisite defects on the Sb site can induce a ferromagnetic interlayer coupling of MnSb_2_Te_4_. Additionally, extrinsic *p*-type doping can further enhance the interlayer ferromagnetic coupling.

## 2. Computational Details

Our first-principles calculations are based on density functional theory [19,20] using the projected plane wave (PAW) method to describe the exchange-correlation energy according to the generalized gradient approximation (GGA) of the Perdew–Burke–Ernzerhof (PBE) type [21]. Compared to PBE calculations, hybrid functionals and self-interaction correction approaches can indeed provide more accurate band structures [22,23]. Nevertheless, GGA (including PBE) calculations provide a reasonably accurate description of the electronic structure of the ground state (including the position of the valence band maximum (VBM)), although they are likely not sufficiently accurate in describing the excited states [24,25]. In this work, the conclusions are reliable as the important formation energies of the defects are based on the fact that the Fermi level of MnSb_2_Te_4_ is around the VBM. The Vienna Ab initio Simulation Package (VASP) is employed to conduct our research [26,27]. We set the cut-off energy to 360 eV and set the energy convergence standard and force convergence standard to 10^−5^ eV and 0.02 eV/Å, respectively. In order to better consider the interaction between layers, we set the vacuum thickness to 16 Å for the slab models. The van der Waals effect is considered with the DFT-D3 method with the Becke–Johnson damping function (D3BJ) [28,29]. We employ a *k*-point mesh centered at the Γ point following the Monkhorst–Pack method, sampling the reciprocal space integral. The Brillouin zone is sampled with an allowed spacing between points of no more than 0.3 Å^−1^. In bulk systems, for example, we employed a 5 × 5 × 2 mesh in the 2 × 2 × 1 supercell model, while we used a 3 × 3 × 2 mesh in the 3 × 3 × 1 supercell model. In the slab model, we employed a 6 × 6 × 1 mesh in the 2 × 2 × 2 slab model, while we used a 4 × 4 × 1 mesh in the 3 × 3 × 2 slab model. We further adopt GGA+U for the Mn 3*d* electrons for the strong correlation effect [30], and the value for U is set to 3 eV according to the earlier literature [31]. The formation energy of intrinsic point defects in MnSb_2_Te_4_ varies with the chemical potentials Δ*μ*_Mn_, Δ*μ*_Sb_, and Δ*μ*_Te_, which can demonstrate the condition from Mn-poor/Sb, Te-rich to Mn-rich/Sb, and Te-poor limits. The chemical potentials are not independent, as follows:

(1) $$\Delta \mu_{\mathrm{Mn}}+2\Delta \mu_{\mathrm{Sb}}+4\Delta \mu_{\mathrm{Te}}=\Delta H({\mathrm{MnSb}}_{2}{\mathrm{Te}}_{4})$$

where Δ*H_f_* is the calculated formation enthalpy of MnSb_2_Te_4_. The stable chemical potential range of MnSb_2_Te_4_ is investigated to avoid possible competing phases of Mn, Sb, and Te. To prevent the formation of binary phases (MnTe, MnTe_2_, SbTe, SbTe_2_, and Sb_2_Te_3_) and elemental species, the following constraints were imposed on the chemical potentials in this work:

(2) $$\Delta \mu_{\mathrm{Mn}}⩽0,\Delta \mu_{\mathrm{Sb}}⩽0,\;\Delta \mu_{\mathrm{Te}}⩽0,$$

(3) $$\Delta \mu_{\mathrm{Mn}}+\Delta \mu_{\mathrm{Te}}⩽\Delta H(\mathrm{MnTe}),$$

(4) $$\Delta \mu_{\mathrm{Mn}}+2\Delta \mu_{\mathrm{Te}}⩽\Delta H({\mathrm{MnTe}}_{2}),$$

(5) $$\Delta \mu_{\mathrm{Sb}}+\Delta \mu_{\mathrm{Te}}⩽\Delta H(\mathrm{SbTe}),$$

(6) $$\Delta \mu_{\mathrm{Sb}}+2\Delta \mu_{\mathrm{Te}}⩽\Delta H({\mathrm{SbTe}}_{2}),$$

(7) $$2\Delta \mu_{\mathrm{Sb}}+3\Delta \mu_{\mathrm{Te}}⩽\Delta H({\mathrm{Sb}}_{2}{\mathrm{Te}}_{3}),$$

where Δ*H*(MnTe), Δ*H*(MnTe_2_), Δ*H*(SbTe), Δ*H*(SbTe_2_), and Δ*H*(Sb_2_Te_3_) represent the formation enthalpies of MnTe, MnTe_2_, SbTe, SbTe_2_, and Sb_2_Te_3_, respectively.

For elemental doping conditions, the chemical potentials are also constrained by the following inequality:

(8) $$\Delta \mu_{\mathrm{Na}}⩽0,\;\Delta \mu_{\mathrm{Mg}}⩽0,\;\Delta \mu_{K}⩽0,\;\Delta \mu_{\mathrm{Ca}}⩽0,$$

(9) $$\Delta \mu_{N}⩽0,\;\Delta \mu_{P}⩽0,\;\Delta \mu_{\mathrm{As}}⩽0,$$

(10) $$\Delta \mu_{\mathrm{Na}}+\Delta \mu_{\mathrm{Te}}⩽\Delta H(\mathrm{NaTe}),$$

(11) $$2\Delta \mu_{\mathrm{Na}}+\Delta \mu_{\mathrm{Te}}⩽\Delta H({\mathrm{Na}}_{2}\mathrm{Te}),$$

(12) $$\Delta \mu_{\mathrm{Mg}}+\Delta \mu_{\mathrm{Te}}⩽\Delta H(\mathrm{MgTe}),$$

(13) $$2\Delta \mu_{K}+\Delta \mu_{\mathrm{Te}}⩽\Delta H(K_{2}\mathrm{Te}),$$

(14) $$\Delta \mu_{\mathrm{Ca}}+\Delta \mu_{\mathrm{Te}}⩽\Delta H(\mathrm{CaTe}),$$

(15) $$\Delta \mu_{\mathrm{Mn}}+\Delta \mu_{\mathrm{Te}}⩽\Delta H(\mathrm{MnTe}),$$

(16) $$\Delta \mu_{\mathrm{Sb}}+\Delta \mu_{N}⩽\Delta H(\mathrm{SbN}),$$

(17) $$\Delta \mu_{\mathrm{Mn}}+\Delta \mu_{P}⩽\Delta H(\mathrm{MnP}),$$

(18) $$\Delta \mu_{\mathrm{Mn}}+\Delta \mu_{\mathrm{As}}⩽\Delta H(\mathrm{MnAs}),$$

(19) $$\Delta \mu_{\mathrm{Sb}}+\Delta \mu_{\mathrm{As}}⩽\Delta H(\mathrm{SbAs}),$$

similarly Δ*H* of compounds represents the formation enthalpies of corresponding compounds.

During the calculation process of intrinsic defect formation energies and the estimation of thermal equilibrium concentration, we perform calculations in three-dimensional structures. For the calculation of interlayer magnetic coupling, we utilize a slab model, which corresponds to a two-dimensional structure.

The energy difference (Δ*E*) between FM coupling (*E*_FM_) and AFM coupling (*E*_AFM_) per Mn atom is calculated to reflect the magnetic coupling strength:

(20) $$\Delta E=E_{\mathrm{FM}}-E_{\mathrm{AFM}}.$$

## 3. Results and Discussion

### 3.1. Intrinsic Defects of MnSb_2_Te_4_ at Low Concentrations

**Figure 1:**
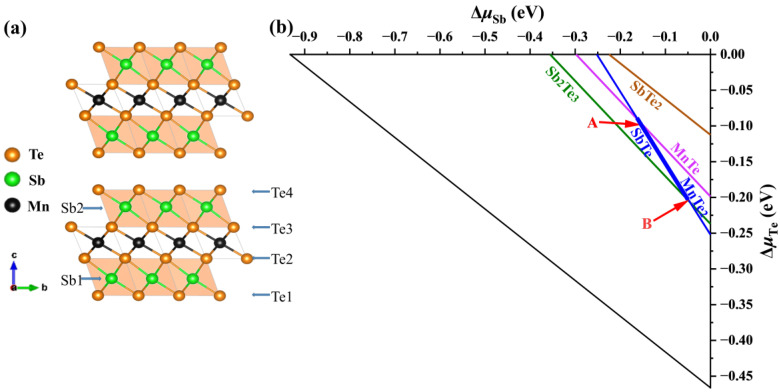
(**a**) Crystal structure of MnSb_2_Te_4_ and (**b**) calculated chemical potential ranges of constituent elements in MnSb_2_Te_4_. The stable regions of MnSb_2_Te_4_ in the phase diagrams (**b**) are represented by the line segment between points A (Δ*μ*_Mn_ = −1.18 eV, Δ *μ*_Sb_ = −0.16 eV, and Δ *μ*_Te_ = −0.09 eV) and B (Δ *μ*_Mn_= −0.95 eV, Δ *μ*_Sb_= −0.04 eV, and Δ *μ*_Te_ = −0.21 eV), which correspond to the Mn-poor and Mn-rich conditions, respectively.

The calculated formation enthalpy of MnSb_2_Te_4_ and MnTe_2_ + 2SbTe → MnSb_2_Te_4_ is close to −0.5 meV. Since its enthalpy is close to 0, the two-phase boundary between ternary phases (MnSb_2_Te_4_) and binary secondary phases (MnTe_2_ and SbTe) will be close. We consider various competing binary compounds, such as MnTe_2_, SbTe, etc. In the phase diagram displayed in Figure 1a,b, the polygon of the chemical potential range calculated under the condition of thermal equilibrium is represented by a thick line segment.

To confirm the chemical potential range, we have performed more precise calculations (the energy convergence standard and force convergence standard are 10^−6^ eV and 0.01 eV/Å, respectively) and find that their deviations are within 3 meV (for Sb and Te). The result shows that MnSb_2_Te_4_ is in a metastable state, and the stable potential range of the thick line segment is almost within the error bar. Nevertheless, we calculate the defect formation energies of intrinsic defects in the Mn-rich and Mn-poor limits (corresponding to points A and B in the figure, respectively) based on the bulk structure. We use the following formula when calculating the defect formation energy [32]:

(21) $$\Delta H_{f}(\alpha ,q)\;=\;E(\alpha ,q)\;-\;E(0)\;+\;\sum_{\alpha }n_{\alpha }(\Delta \mu_{\alpha }+\mu_{\alpha })\;+\;q(E_{\mathrm{VBM}}+E_{F}),$$

where *E*(*α*, *q*) represents the energy of a defect *α* with a charge state of *q*, *E*(0) represents the energy of a supercell without defects, *n* represents the number of defects *α*, and *μ_α_* represents the chemical potential of the element. *E*_VBM_ represents the energy at the top of the valence band when there is no defect, and *E*_F_ is the Fermi energy relative to *E*_VBM_.

**Figure 2:**
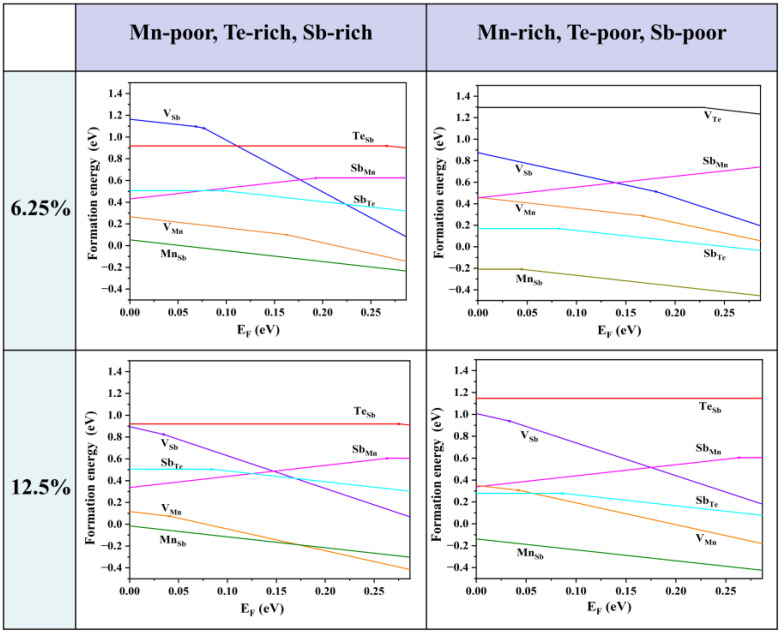
Calculated formation energies of intrinsic defects as a function of the Fermi level (variable from the VBM to the CBM) in MnSb_2_Te_4_ at the Mn-poor and Mn-rich limits, which correspond to points A and B in Figure 1b. The plots in the first row are calculated in a 2 × 2 × 2 supercell size with one defect (i.e., 6.25% Mn antisite defects), and the plots in the second row are calculated in a 2 × 2 × 1 supercell size with one defect (i.e., 12.5% Mn antisite defects). The slope of defect formation energy shows the charge state of the defect.

Firstly, we consider the formation energies of various intrinsic defects at different chemical potentials. Using Equation (21), we calculate the formation energies of all the intrinsic defects in their relevant states in MnSb_2_Te_4_. Of note, the Makov–Payne image charge correction [33] is not adopted here due to the relatively high defect concentration in the system, i.e., the simulated defect concentrations are close to the practical values. According to earlier reports, there are a large number of intrinsic defects in MnSb_2_Te_4_, including vacancies, antisite defects, and other forms. In Figure 2, we show the defect formation energies of the Mn-poor/rich chemical potential limit conditions. The Fermi level is tuned between the valence band maximum (VBM) and the conduction band minimum (CBM). The defect formation energies are shown as a function of Fermi level at the Mn-poor limit and the Mn-rich limit. The lines represent the charge state with the lowest formation energy in the energy range. The slope of the lines represents the charge state of the defect, and the kinks between the line segments denote the transition of the charge state. From Figure 2, we find that at the Mn-rich/Sb, Te-poor limit condition, the charge state of Mn antisite defects undergoes a transition from 0 to −1 in this energy range at 6.25% concentration, while the charge state of Mn antisite defects is maintained at −1 in the Mn-poor/Sb, Te-rich limit. From a Mn-poor condition to a Mn-rich condition at 6.25% concentration, the formation energy of the Mn antisite defect decreases from 0.05 eV to −0.21 eV. The results show that the chemical environment has a great influence on the formation energy and the charge state of different defects. With consideration of the charge neutrality condition, it is determined that the Fermi level is close to the VBM, and thus MnSb_2_Te_4_ has a *p*-type conductivity intrinsically.

As the Fermi level is located near the VBM, the formation energy near the VBM is taken into account for comparison. The formation energies of the Mn antisite defects are always low, no matter if under Te-poor or Te-rich conditions, indicating that Mn_Sb_ is the most abundant intrinsic defect in MnSb_2_Te_4_. Sb_Mn_ is the most stable donor, while V_Mn_, V_Sb_, and Mn_Sb_ are acceptors. Importantly, the formation energy of the Mn antisite defect is negative at low concentrations (<6.25%), indicating the spontaneous formation of the defect in pristine systems, which is consistent with theoretical results in the literature [15]. The formation energy of Mn_Sb_ at 12.5% (−0.01 eV) is greater than the formation energy at 6.25% (−0.17 eV). Therefore, it is expected that the formation energy of Mn_Sb_ increases as the concentration increases and turns into positive values beyond a certain concentration.

### 3.2. Defect Concentration of Mn Antisite Defect under a Thermal Equilibrium

**Figure 3:**
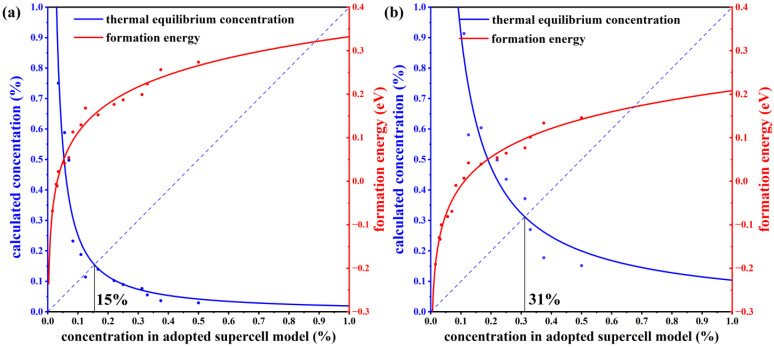
The thermal equilibrium concentration of Mn antisite defects. The red fitting curve represents the calculated defect formation energy of Mn_Sb_ from our adopted supercell model with the corresponding concentration. The blue fitting curve represents the calculated defect concentration according to the calculated defect formation energy under thermodynamic equilibrium conditions. Plots (**a**,**b**) correspond to Mn-poor/Sb, Te-rich, and Mn-rich/Sb, Te-poor conditions, respectively.

To verify the stability of the structure, we have calculated the defect formation energies at different concentrations. The charge neutral condition is always required in the calculation of the formation energy. We calculate the formation energy of Mn antisite defects in different supercells with 14, 42, 56, 98, 126, and 224 atoms, whose corresponding configurations are displayed in Figure S1 of the Supplemental Materials. The lowest defect formation energy from multiple configurations at each concentration is adopted to calculate the thermal equilibrium concentration, as shown in Figure 3. For instance, at a 33.3% concentration in Mn-poor/Sb and Te-rich conditions, 0.18 eV and 0.22 eV are obtained, respectively, and we chose 0.18 eV as the formation energy in Figure 3. We have performed calculations on the formation energies of defects with and without SOC and observed a consistent trend of variation with concentration. Upon considering SOC, the overall defect formation energy has a notable elevation of approximately 0.05 eV. Due to the significant spin-orbit coupling interaction of the Mn element, we have specifically chosen to compute the formation energy of defects considering spin-orbit coupling, aiming to obtain a more precise estimation of the defect equilibrium concentration under thermal equilibrium. The defect concentration is calculated by:

(22) $$n=N_{D}\exp(-\Delta H_{f}/k_{B}T),$$

where *N*_D_ is the concentration of available atomic sites for defect formation, Δ*H_f_* is the defect formation energy, *k*_B_ is the Boltzmann constant, and *T* is temperature (here, the melting point of MnSb_2_Te_4_ of 918 K [17] is adopted). We calculate the defect formation energy from the concentration of a series of structures and then estimate the thermal equilibrium concentration of Mn antisite defects at Sb sites based on Equation (22). As displayed in Figure 3, Mn antisite defects at the Sb site are easier to form when the defect concentration is low but difficult to form when the concentration is high, indicating that Mn antisite defects will not accumulate forever and will reach thermal equilibrium at a certain concentration. In this way, an intersection point can be found between the concentration in an adopted supercell model and the thermodynamic equilibrium concentration, which indicates the state of thermodynamic equilibrium. From Figure 3a,b, the chemical potential greatly affects the formation of defects. The equilibrium concentration of Mn antisite defects is 15% under Mn-poor/Sb and Te-rich conditions, while its concentration is 31% under Mn-rich/Sb and Te-poor conditions, consistent with the experimental report that concentrations of Mn antisite defects are to be 15% [16] or 19.3% [17].

**Figure 4:**
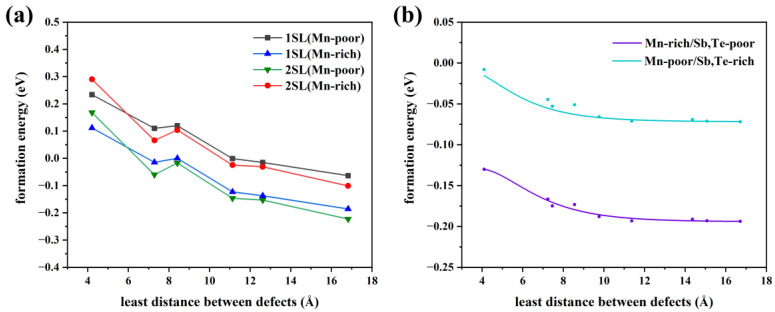
(**a**) The relationship between defect formation energy of Mn antisite defect and distance at different concentrations (only one defect in each supercell model), 1SL/2SLs in the figure means that the supercell size of 1/2 septuple layer is selected in the calculation. (**b**) The relationship between the defect formation energy of a Mn antisite defect and the distance between defects at a concentration of 3.13% (two defects in a 4 × 4 × 2 supercell model).

Consequently, we will discuss the influence of the defect distribution on the defect formation energy. In Figure 4b, the relationship between the various distributions of Mn antisite defects at a 6.25% concentration and formation energy is displayed. The larger the distance between the defects, the lower the formation energy, whether in Mn-poor or Mn-rich conditions, revealing a tendency for Mn antisite defects to be uniformly in MnSb_2_Te_4_. When the nearest neighbor distance reaches 12 Å, the defect formation energy, tends to converge. Additionally, various supercell models at 1SL and 2SLs MnSb_2_Te_4_ with only one defect are calculated to facilitate the comparison of defect distribution (Figure 4a). The formation energy decreases as the distance to the nearest neighbor increases, in line with the above observation. In Figure 4b, the variation of formation energy is small in the case where only the influence of defect distribution is considered. For example, under Mn-poor conditions, the defect formation energy only decreases from −0.02 eV to −0.06 eV, which is much smaller than the case in Figure 4a. As a result, concentration has a more significant impact than distribution on the formation energy of defects.

**Figure 5:**
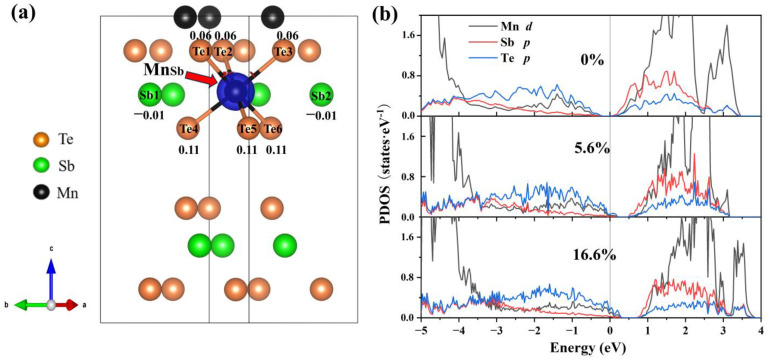
Differential charge density and projected density of states (PDOS) of MnSb_2_Te_4_. (**a**) The differential charge density of MnSb_2_Te_4_ with a 16.6% Mn antisite defect. The blue color represents charge depletion. The value around atoms represents the number of electrons obtained by the atom. The positive and negative values represent charge depletion and charge accumulation, respectively. Projected density of states (PDOS) of MnSb_2_Te_4_ with Mn antisite defects at different concentrations of (**b**) 0%, 5.6% and 16.6%.

To explain this trend in the formation of energy, we analyze the electronic structure at various concentrations. From Figure 5a, the neighboring Te atoms around the Mn antisite defect exhibit a significant charge accumulation (0.06 to 0.11), indicating that the introduction of Mn antisite defects primarily alters the electronic distribution of Te. The interlayer van der Waals interactions result in a more pronounced accumulation of charge in the Te atoms closer to the van der Waals layers. In the PDOS (Figure 5b), Te-*p* orbitals dominate the variation of the density of states near the VBM. The ground state (Figure 5b) in which the Fermi level is in the ground state is between the conduction band and the valence band. Comparing the PDOS diagrams, as the concentration increases, the Fermi level gradually moves to the valence band, which is related to the spin polarization of Te-*p* orbitals. It is clear that the Mn antisite defect is a deep acceptor.

### 3.3. Magnetic Interlayer Coupling of MnSb_2_Te_4_ with Mn Antisite Defects

**Figure 6:**
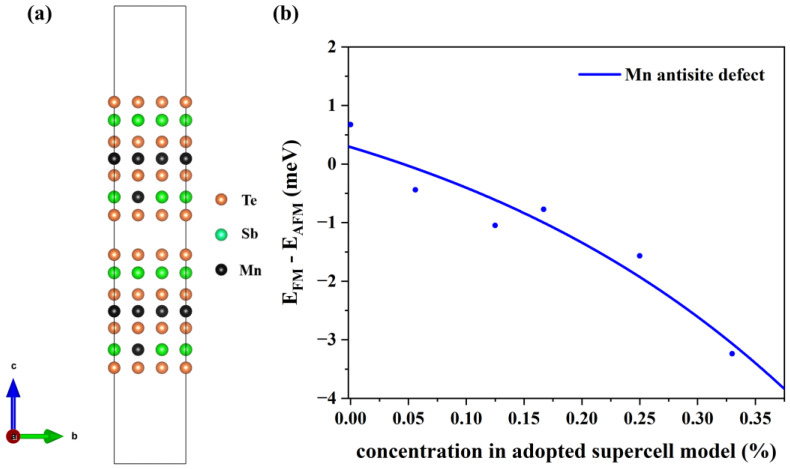
(**a**) The structure of MnSb_2_Te_4_ with Mn_Sb_ at a 16.6% concentration. (**b**) The energy differences between the interlayer ferromagnetic and interlayer non-ferromagnetic states in 2SLs MnSb_2_Te_4_ at different concentrations for Mn_Sb_.

Through the above discussion, the Mn antisite defect can reach an equilibrium state within a concentration range of 15–31%. Next, we calculate the effect of these defects on the interlayer coupling with different supercell sizes in slab models. In 2SLs MnSb_2_Te_4_, there are two defects in each supercell (e.g., Figure 6a). As displayed in Figure 6b, Mn antisite defects can tune the MnSb_2_Te_4_ interlayer coupling from an ideal AFM state to an FM state. The FM interlayer coupling is enhanced with increasing concentrations. When the concentration of Mn antisite defects reaches 33%, the energy difference between the FM state and the AFM state is up to −3.2 meV/Mn. Our results demonstrate that Mn antisite defects can induce a transition in interlayer magnetic coupling, which has been mentioned in previous experiments [16,34] in that Mn-Sb site mixing can cause such transitions. In contrast to previous studies, our research focuses more on whether individual Mn antisite defects alone can induce this magnetic phase transition. This confirms that Mn antisite defects correspond to the observation of FM states in MnSb_2_Te_4_.

**Figure 7:**
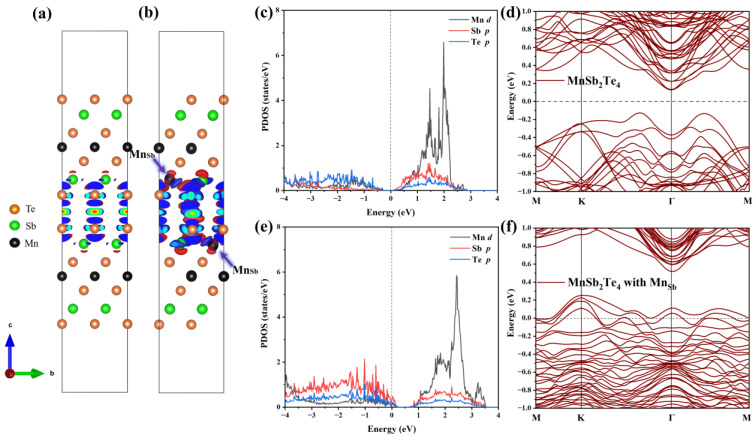
Differential charge density of (**a**) pristine 2SLs MnSb_2_Te_4_ and (**b**) 2SLs MnSb_2_Te_4_ with Mn antisite defects. The blue- and red-colored isosurfaces represent charge depletion and charge accumulation, respectively. Projected density states of pristine 2SLs MnSb_2_Te_4_ and 2SLs MnSb_2_Te_4_ with Mn antisite defects are illustrated in (**c**,**e**). Band structures without SOC of (**d**) pristine and (**f**) 2SLs MnSb_2_Te_4_ with Mn antisite defects at a concentration of 12.5% with optimal configurations along high-symmetry lines.

Next, we analyze the internal mechanism of this ferromagnetic coupling. The differential charge density is displayed in the case of no defects and in the case of Mn antisite defects, as shown in Figure 7a,b. In the absence of defects, the charge distribution in the layered structure of MnSb_2_Te_4_ is relatively symmetrical, and the charge distribution in the upper SL and the lower SL in 2SLs is almost consistent. However, in the case of Mn antisite defects, the charge concentrates near Mn and is greatly changed in the lower and upper SLs. The charge distribution caused by Mn antisite defects leads to a change in the orbital occupation states of Mn and Te. Figure 7c–f displays the densities of state and band structure for pristine 2SLs MnSb_2_Te_4_ and 2SLs MnSb_2_Te_4_, respectively, at the concentration of Mn antisite defects of 12.5%. The peak of the *p* orbital of Te atoms gradually approaches the Fermi level with Mn antisite defects, while the peaks of the *d* orbitals of Mn atoms also shift right. This indirectly indicates that the interlayer ferromagnetism is related to Te-Mn-Te superexchange interactions. Comparing 2SLs MnSb_2_Te_4_ and 2SLs MnSb_2_Te_4_ with Mn antisite defects in the slab model, we conclude Mn antisite defects exhibit a deep acceptor behavior, which is consistent with the case of the bulk system.

Since intrinsic defects can induce magnetic transitions, we wonder whether external doping will have an additional influence on the system. According to previous research, *p*-type doping in MnBi_2_Te_4_ affects the occupation state of the *d* orbitals of Mn atoms, resulting in unusual long-range superexchange interactions mediated by the *p* orbitals across the vdW gap, and thus indirectly affecting the interlayer coupling [9]. In this way, the interaction between the 3*d* orbitals of the SL layers without doping elements and other SL layers doped with *p*-type elements can promote the interlayer coupling from AFM to FM. The high concentration of doping (44% at the Sb site) of Cr in Sb_2_Te_3_ promotes its Curie temperature to 250 K from 80 K (12% at the Sb site) [35]. The result reflects that the influence of doping elements on magnetic properties is remarkable. The fact that Sb_2_Te_3_ was successfully doped by a Cr element in the experiment provides a feasible solution to tuning the interlayer coupling.

### 3.4. Magnetic Interlayer Coupling with Additional p-Type Doping

In addition to controlling the defect concentration, *p*-type doping defects are predicted to regulate the magnetic properties of MnBi_2_Te_4_ [36]. We consider the *p*-type doping in the two-dimensional MnSb_2_Te_4_ structure, such as Sb substituted by Na/Mg/K/Ca or Te substituted by N/P/As. As shown in Figure 1a, *p*-type doped Sb and Te mainly have two and four types of sites, respectively. The formation energy of the 2SLs structure is calculated as a function of the chemical potential of the Sb/Te element.

**Figure 8:**
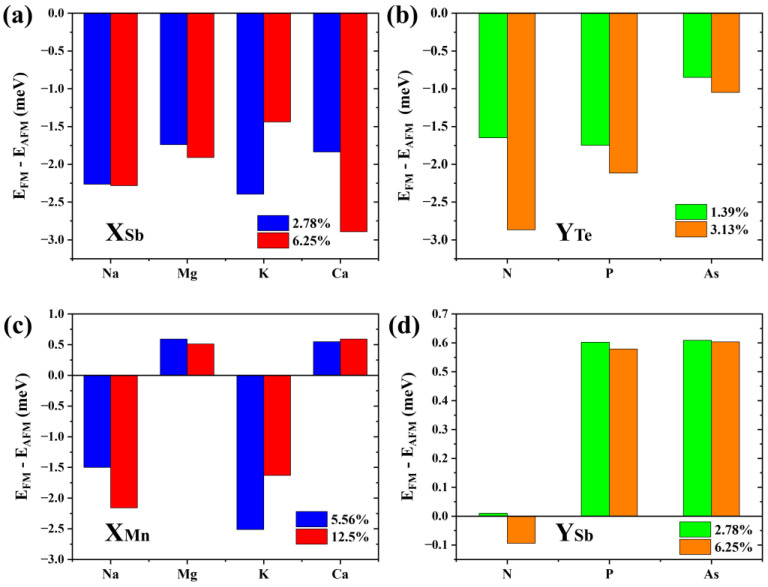
The energy differences between interlayer ferromagnetic and interlayer antiferromagnetic states in (**a**) Na/Mg/K/Ca doped 2SLs MnSb_2_Te_4_ at concentrations of 2.78% and 6.25%; (**b**) N/P/As doped 2SLs MnSb_2_Te_4_ at concentrations of 1.39% and 3.13%, (**c**) Na/Mg/K/Ca doped 2SLs MnSb_2_Te_4_ at concentrations of 5.56% and 12.5%; and (**d**) N/P/As doped 2SLs MnSb_2_Te_4_ at concentrations of 2.78% and 6.25%. X and Y represent Na/Mg/K/Ca and N/P/As, respectively.

Results show that the formation energy of the Na-substituted Sb2 site (about −0.6 eV to −0.5 eV) is lower than that of other *p*-type doping elements (Mg: −0.1 eV to 0 eV; K: 0.32 eV to 0.42 eV; and Ca: 0.23 eV to 0.33 eV). The defect formation energy of the N-substituted Te4 site (about 0.83 eV to 0.98 eV) is significantly higher than that of P (about 0.41 eV to 0.52 eV) and As (about 0.1 eV to 0.2 eV). Of note, the low (even negative) formation energies of these dopants are largely due to the meta-stability of the host material, and the charge states of these defects are not considered following the charge neutrality condition. Figure 8a,b show the energy difference between interlayer FM and AFM coupling of 2SLs MnSb_2_Te_4_ under different defect concentrations. Apparently, MnSb_2_Te_4_ shows interlayer ferromagnetism with different *p*-type dopants. For instance, for N atoms doped on the Te site, interlayer coupling strengthens from −1.6 to −2.9 meV/Mn with increasing N_Te_ concentration.

In addition, we consider other doping possibilities. We calculate the formation energy of the bilayer septuple layer structure at a Te-poor condition. Results show the formation energy of the Na, Mg, K, and Ca-substituted Mn sites is about −1.35 eV, −1.6 eV, −1.1 eV, and −2.8 eV, respectively. The defect formation energies of the N, P, and As-substituted Te4 sites are about 2.6 eV, 0.6 eV, and 0.1 eV, respectively. As displayed in Figure 8c,d, when Na or K is doped, the interlayer coupling of MnSb_2_Te_4_ also becomes ferromagnetic, while MnSb_2_Te_4_ exhibits interlayer antiferromagnetic coupling when doped with Mg or Ca. This shows that *p*-type doping can effectively regulate the interlayer coupling of MnSb_2_Te_4_. Element replacement in the same valence state does not significantly affect the interlayer coupling of MnSb_2_Te_4_. Similarly, we also calculated the cases where N, P, and As replaced the Sb position and no apparent interlayer FM was induced. This further verifies the rationality of *p*-type doping.

## 4. Conclusions

In summary, we have studied the stability of MnSb_2_Te_4_ and its intrinsic defects by first-principles calculations. We demonstrate that the most stable defect among them is the Mn antisite defect, which is easy to form under Mn-rich conditions. Mn antisite defects will be at a rather high concentration of 15% at a Mn-poor/Sb, Te-rich condition or 31% at a Mn-rich/Sb, Te-poor condition in typical MnSb_2_Te_4_ samples under thermal equilibrium. Interestingly, it can turn the interlayer coupling FM from the AFM state of a pristine MnSb_2_Te_4_ system. Furthermore, we demonstrate a *p*-type doping strategy to enhance the interlayer ferromagnetism in MnSb_2_Te_4_.

## Data Availability

The datasets used during the current study are available from the corresponding author upon reasonable request.

## Supplementary Materials

The following supporting information can be downloaded at: https://www.mdpi.com/article/10.3390/ma16155496/s1, Figure S1: Structures for the various supercell sizes, with the concentration of MnSb presented in each configuration.
