# Supplementary material for: High Concentration Intrinsic Defects in MnSb2Te4
Source: Materials (Basel). 2023 Aug 7;16(15):5496. doi: 10.3390/ma16155496 (PMC10420118; doi:10.3390/ma16155496)
Supplement: Supplementary file 1 [file materials-16-05496-s001.zip › materials-2478495-supplementary.pdf]

materials

Article

High Concentration Intrinsic Defects in $MnSb_2Te_4$

Jie Xiong, Yin-Hui Peng, Jia-Yi Lin, Yu-Jie Cen, Xiao-Bao Yang and Yu-Jun Zhao *

Department of Physics, South China University of Technology, Guangzhou 510640, China; 17354426785@163.com (J.X.); pengyinhui88@gmail.com (Y.-H.P.); h752917623h@163.com (J.-Y.L.); cenyjie@gmail.com (Y.-J.C.); scxbyangscut@scut.edu.cn (X.-B.Y.)

* Correspondence: zhaoyj@scut.edu.cn

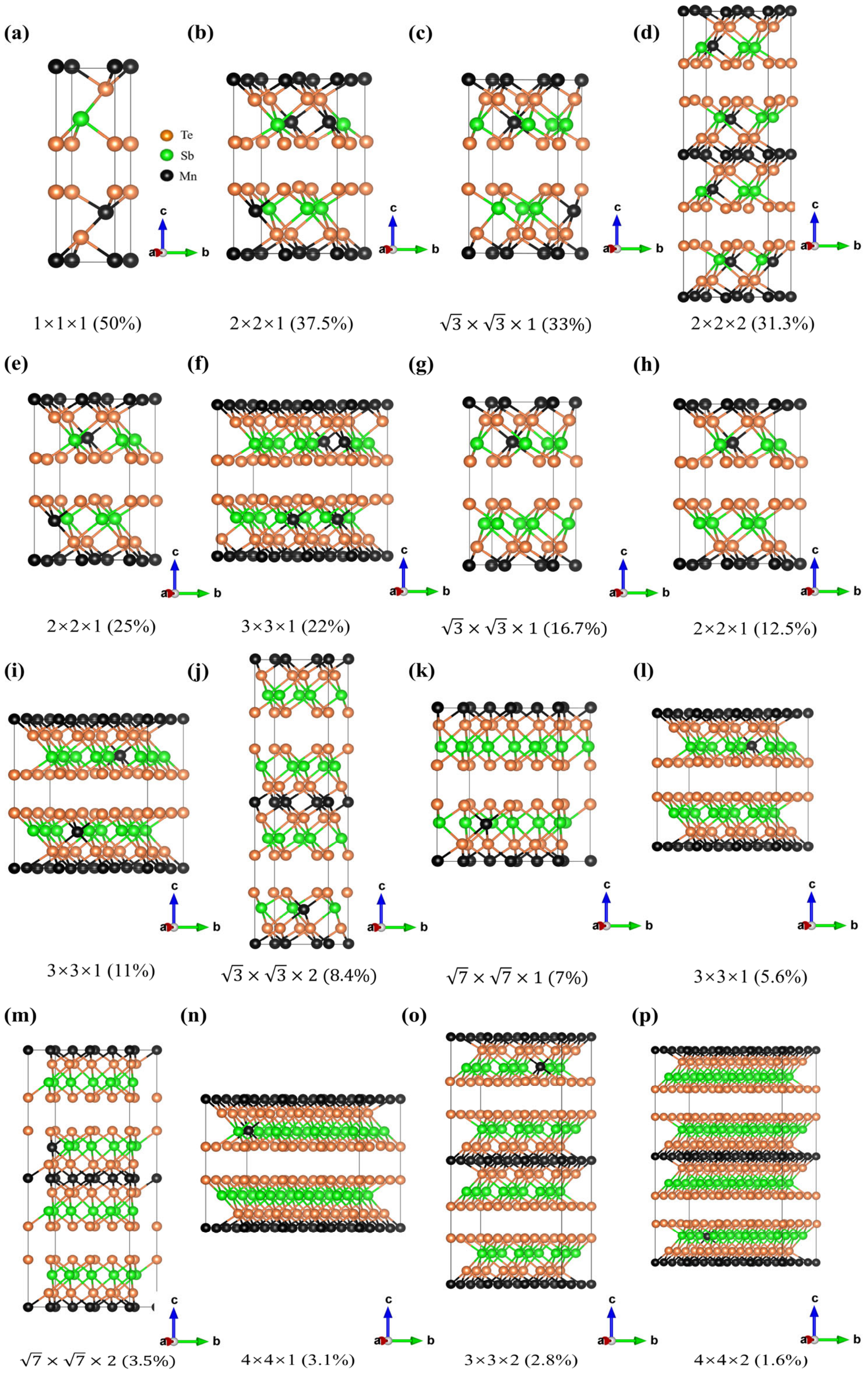

Figure S1. Structures for the various supercell sizes, with the concentration of Mn_{Sb} presented in each configuration.

Figure S1 displays various supercell structures with defects, annotated with their corresponding supercell sizes. All structures in the figure represent the lowest energy configurations obtained from our calculations at the respective concentrations. Black, green, and orange spheres represent Mn, Sb, and Te atoms, respectively.
